# Estimating hospital catchments from in-patient admission records: a spatial statistical approach applied to malaria

**DOI:** 10.1038/s41598-020-58284-0

**Published:** 2020-01-28

**Authors:** Victor A. Alegana, Cynthia Khazenzi, Samuel O. Akech, Robert W. Snow

**Affiliations:** 1 Kenya Medical Research Institute - Wellcome Trust Research Programme, P.O. Box, 43640-00100 Nairobi, Kenya; 2 Geography and Environmental Science, University of Southampton, SO17 1BJ Southampton, UK; 3 Faculty of Science and Technology, Lancaster University LA1 4YR Lancaster, UK; 4 Centre for Tropical Medicine and Global Health, Nuffield Department of Clinical Medicine, University of Oxford, OX3 7LJ Oxford, UK

**Keywords:** Infectious diseases, Computational biology and bioinformatics, Health care, Medical research, Mathematics and computing

## Abstract

Admission records are seldom used in sub-Saharan Africa to delineate hospital catchments for the spatial description of hospitalised disease events. We set out to investigate spatial hospital accessibility for severe malarial anaemia (SMA) and cerebral malaria (CM). Malaria admissions for children between 1 month and 14 years old were identified from prospective clinical surveillance data recorded routinely at four referral hospitals covering two complete years between December 2015 to November 2016 and November 2017 to October 2018. These were linked to census enumeration areas (EAs) with an age-structured population. A novel mathematical-statistical framework that included EAs with zero observations was used to predict hospital catchment for malaria admissions adjusting for spatial distance. From 5766 malaria admissions, 5486 (95.14%) were linked to specific EA address, of which 272 (5%) were classified as cerebral malaria while 1001 (10%) were severe malaria anaemia. Further, results suggest a marked geographic catchment of malaria admission around the four sentinel hospitals although the extent varied. The relative rate-ratio of hospitalisation was highest at <1-hour travel time for SMA and CM although this was lower outside the predicted hospital catchments. Delineation of catchments is important for planning emergency care delivery and in the use of hospital data to define epidemiological disease burdens. Further hospital and community-based studies on treatment-seeking pathways to hospitals for severe disease would improve our understanding of catchments.

## Introduction

Millions of children continue to be infected with *Plasmodium falciparum* every year across sub-Saharan Africa^1,2^. It is estimated that 1-2% of these infections progress to severe, life-threatening morbid complications^3^. Two potentially fatal outcomes of infection in childhood include cerebral malaria and severe malaria anaemia^4–6^. It is unlikely that children who develop these two syndromes would survive in the absence of emergency treatment and supportive interventions provided through in-patient hospital care. For those who reach the hospital, case-fatalities for cerebral malaria remain over 20%^7,8^, and for severe anaemia without blood transfusion over 50%^8,9^. In-patient hospital survival depends crucially on early presentation and immediate triage and intervention^10,11^.

Optimizing timely hospital access is a critical intervention for many emergency care conditions that affect large parts of Africa including those targeting reductions in maternal and neonatal mortality and road accidents^12–14^. These initiatives have led to a range of new approaches for measuring the geographic accessibility of hospital care^15–17^.

Previous hospital-based studies have used physical distance or travel time^18–21^, provider and case ratios^15^, and spatial interaction methods^16^ to define access to hospital. However, the use of distance or travel-time have not explicitly or implicitly modelled competition for services provided by other hospitals in the neighbourhood. For example, the case-ratio method is confined to selected geographic areas, assuming a uniform use of hospital within the selected geographic units^22^. Gravity based models combine aspects of distance-decay and health system characteristics (e.g. hospital size) but do not always include all aspects of population density and demand^17^. In summary, these approaches remain mechanistic and a robust statistical approach improving on these descriptions and integrating disease-specific admission records, mechanistic estimates on distance, population and health system factors to characterize hospital use is required.

Here we employ a mathematical-statistics approach to predict malaria-related hospital access using information recorded at admission at major level 4 and level 5 hospitals. The approach integrates clinical malaria case admissions at the hospital level, fine-spatial resolution mechanistic estimates of distance and travel times, and demographic data among malaria patients reaching the hospitals. A Bayesian geostatistical framework is employed accounting for the spatial variability in admissions related to malaria due to variation in care-seeking at community level^23–25^ as well as a spatial variation in malaria risk^26^. The method also improves on the previous mechanistic distance or travel-time approaches by incorporating population and the spatial dependencies in the admission rates at the community level as well as measures of uncertainty during catchment predictions. Results present a description of catchments representative of malaria care-seeking, and statistical spatial association with severe malaria cases.

## Results

### Malaria admissions at the four hospitals

Table 1 summarises malaria admission at four county referral hospitals. Across two years of observation, there were 5766 inpatient malaria admissions for children 1 month to 14 years. It was possible to define a specific EA address for 5486 (95.14%) malaria admissions. Of the geocoded cases, 4972 (90.6%) had complete information for CM classification while all the 5486 (100%) had information on SMA. 272 were CM based on children who were unconscious (U) or could only respond to a painful stimulus (P) (AVPU = P or U) while 1001 were SMA based on the combination of haemoglobin ≤5 g/dL and clinical features (severe pallor or blood transfusion). Out of the 5,766 records investigated, retrospectively, 194 (3.4%) were not geocoded or linked to an EA address. Of the 194, information was only available for 76 records (71, were from Uganda (associated with Busia county referral hospital and 5 from Kisumu county referral hospital). There was no EA information to geocode address for the cases from Uganda. The Kisumu cases were more than 200 km from the Kisumu County hospital (Nandi county (3), Kisii County (4), and Kericho county (1). For the other cases, 3 were for adults while 115 were missing.

**Table 1:** Descriptive summary of inpatient malaria admissions for the number of children 1 month to 14 years at hospital level and the severe malaria admissions (cerebral (CM) and severe malarial anaemia (SMA)).

Hospital	Busia County Referral Hospital	Kakamega County Teaching and Referral Hospital	Kisumu County Referral Hospital	Vihiga County Referral Hospital	Total
**Cerebral Malaria**					
Number malaria admissions with CM information Georeferenced to EA	1352	1856	1214	550	4972
Defined CM cases Georeferenced to EA	79	75	45	73	272
CM cases in predicted catchment	52	37	28	6	123
**Severe Malaria Anaemia**					
Number of malaria admission with SMA information Georeferenced to EA	1567	1939	1353	627	5486
Defined SMA cases Georeferenced to EA	374	417	133	77	1001
SMA cases in predicted catchment	194	178	72	9	453

IQR: Inter-quartile range; CM: Cerebral Malaria; SMA: Severe Malaria Anaemia.

### Estimation of hospital catchments from malaria admissions

The estimation of hospital catchment area was conducted using EAs encompassing geocoded malaria admissions. It was assumed that the likelihood of admission varied spatially. A Bayesian hierarchical zero-inflated Poisson spatial regression model was fit to the observed counts (including zero) at the EA (using EA centroid) to analyse admission rates using an underlying population for children between 1 month to 14 years. Thus, each EA was assumed to either generate no malaria admissions (zeros), a single malaria admission, or, a set of several admissions (excluding the CM and SMA cases subset for subsequent catchment analysis). EAs with zero admissions arose if there was no malaria case admitted to the study hospital (an actual zero) or malaria case(s) was present but the patient(s) opted to use a different hospital within the study area. Therefore, the novelty of the modelling approach here included aspects of competition implicitly by including zero counts combined with population and spatial ancillary data. It was not possible to include a hospital competition parameter explicitly at the EA level because geocoded admissions data from the competing hospitals were not available. The Bayesian specification included covariate (distance to the nearest road) and the model goodness of fit was assessed based on the deviance information criterion (DIC). Further, the assessment of model performance such as the root mean square error (RMSE) was based on a 30% subset of data. To avoid methods induced circularity in the subsequent analysis of how SMA and CM admission rates corresponded to the predicted malaria catchments, the severe malaria admissions were excluded from the estimation of general malaria catchments. Further details of the Bayesian model specification are included in the methods section.

**Figure 1:**
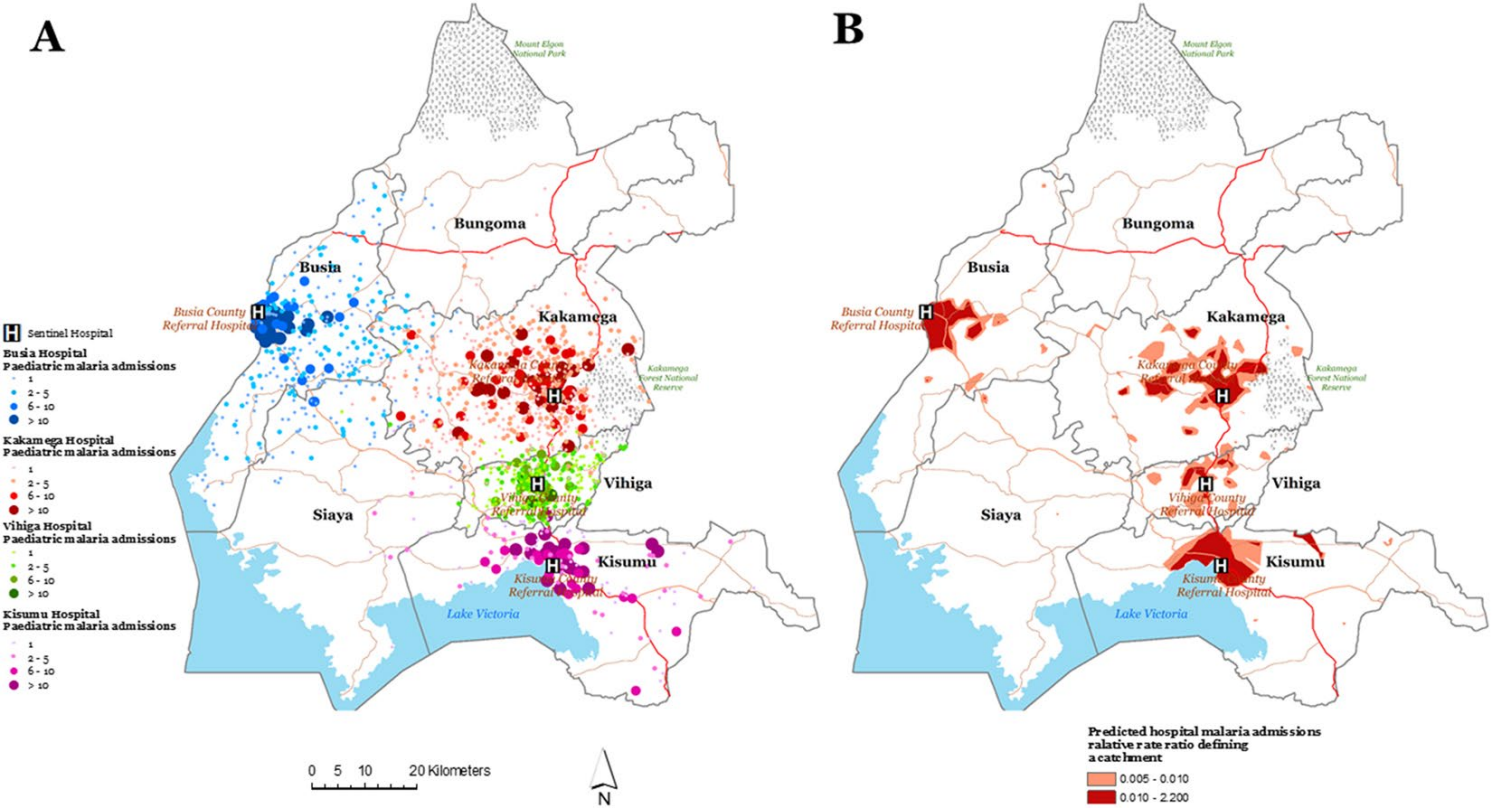
(**A**) Map showing the spatial distribution (counts) of all inpatient malaria admissions georeferenced to an EA address for children between 1 month and 14 years at the county referral hospital in western Kenya (Busia hospital *n* = 1140 admissions), Kakamega hospital (*n* = 1471 admissions), Vihiga hospital (*n* = 486 admissions) and Kisumu hospital (*n* = 1184 admissions). Data exclude severe malaria cases (SMA and CM cases). (**B)** The Bayesian predicted posterior median rate-ratio of hospital use (admission) for malaria defining a catchment by hospital adjusting for population, distance to the hospital. The prediction of catchment areas excluded CM and SMA cases.

Figure 1A shows the spatial distribution of malaria admissions (*n* = 4,281) excluding the severe cases (SMA and CM) at the four sentinel hospitals. Figure 1B shows the delineated catchments representing malaria admissions based on the Bayesian predicted posterior median. The geographic extent of the catchments varied by hospital i.e. 168.1 km^2^ in Busia, 322.0 km^2^ in Kakamega, 24 km^2^ in Vihiga and 204.0 km^2^ in Kisumu. Figure 1B shows the malaria admissions catchments were not homogenous around the hospital, did not cover the entire health administrative boundary (i.e. the county), and were not of distinct geometrical shape. This spatial variation suggested either a variation in hospital use for malaria care-seeking at the level 4/5 hospital or in the risk of developing severe malaria.

### Sensitivity analysis for hospital catchments

Several candidate models were considered in the Bayesian paradigm based on mean deviance, the DIC, the WAIC, the RMSE and the proportion of variance explained by the candidate model. Table 2 list best model fitting parameters for models selected minimizing the DIC, WAIC, MLS whilst exhibiting the best performance in terms of the proportion of variance explained while Table S1 (in the Supplementary Information) shows a combination of these parameters for the wider candidate models. Model performance improved with a refinement of the Delaunay triangulation of the spatial region considered. The proportion of variance explained by best candidate model based on the observed inpatient paediatric admissions was highest in Vihiga (90%) compared to Kakamega (74%), Busia 55%, and 56% for Kisumu (Table 2).

**Table 2:** Bayesian model selection parameters for catchment prediction using malaria admissions. The goodness of fit parameters applies to the best fitting model for each sentinel hospital. The extended model selection results are included in the Supplementary Information as Table S1.

Hospital	PD	DIC	WAIC	MLS	RMSE	Fraction of variance explained
Busia County Referral Hospital	85.02	3597.62	3937	0.91	0.0015	55.07
Kakamega County Teaching and Referral Hospital	58.52	5757.3	5975.1	1.08	0.0018	75.72
Kisumu County Referral Hospital	22.28	2283.91	2344.47	0.71	0.0664	56.52
Vihiga County Referral Hospital	40.81	1323.63	1373.55	1.01	0.0026	91.01

### Spatial determinants for severe malaria admissions

For all severe malaria admissions, the median straight-line distance to the admitting level 4 or 5 hospital was 0.98 km (interquartile range 0.47–1.96 Km) for CM and this was 0.90 km (interquartile range 0.43–1,64 Km) for SMA. Overall, the corresponding predicted median travel times to the four hospitals was 38.5 minutes (interquartile range 20.51–55.76 minutes) for CM and 45.9 minutes (interquartile range 22.41–70.99 minutes) for SMA, assuming optimal travel without delays. Distances and travel times however varied between hospitals (Table S2 in the Supplementary Information).

Table 3 shows the Bayesian posterior estimates for spatial determinants of CM and SMA for each hospital. The individual-level model for CM and SMA described the rates of admission adjusting for travel time to the hospital, the spatial location of a case within compared to those outside the predicted catchment, age (as a continuous random effect), and seasonality (month).

**Table 3:** Summary of results on spatial determinants for cerebral malaria (CM) and severe malaria anaemia (SMA) admissions at the four sentinel hospitals. The table shows regression effects (the posterior median rate ratios and the 95% Bayesian Credible Interval) based on geographic access characteristics of travel time and predicted catchments from inpatient malaria areas. The model adjusted for random effects on the age of child, the month of admission (random effect), travel time to the hospital, and a spatial effect based on child EA.

Variable	Busia County Referral Hospital	Kakamega County Teaching and Referral Hospital	Kisumu County Referral Hospital	Vihiga County Referral Hospital
	Median (95% CrI)	Median (95% CrI)	Median (95% CrI)	Median (95% CrI)
**Cerebral malaria cases**				
**Catchment**				
Inside predicted catchment	1.00	1.00	1.00	1.00
Outside predicted catchment	0.31 (0.15–0.59)	1.02 (0.6–1.59)	0.59 (0.24–1.27)	0.67 (0.24–1.4)
**Travel time to hospital**				
<10 minutes	1.00	1.00	1.00	1.00
10–30 minutes	0.67 (0.36–1.16)	0.81 (0.22–3)	1.99 (0.64–5.97)	1.63 (0.58–4.37)
30–1 hour	0.29 (0.11–0.61)	1.61 (0.47–5.88)	4.17 (1.32–12.68)	3.09 (1.13–8.18)
>1 hour	0.17 (0.06–0.43)	2.13 (0.6–7.95)	1.1 (0.15–5)	2.63 (0.81–7.65)
**Random effects**				
Month of admission (Seasonal variance parameter)	0.06 (0–0.91)	1.22 (0.34–3.74)	0.54 (0.2–1.32)	0.63 (0.23–1.61)
Age (Variance parameter)	0 (0–0)	0 (0–0)	0.11 (0–0.52)	0.01 (0–0.05)
Spatial variance	0.07 (0–0.82)	0.07 (0–0.98)	0.65 (0.06–3.02)	0.05 (0–0.52)
Spatial range (Decimal degree)	0.02 (0–0.08)	0.03 (0.01–0.08)	0.09 (0.03–0.29)	0 (0–0.02)
**Severe Malaria Anaemia**				
**Catchment**				
Inside predicted catchment	1.00	1.00	1.00	1.00
Outside predicted catchment	0.34 (0.18–0.55)	0.77 (0.51–1.09)	0.53 (0.25–1)	1.09 (0.42–2.29)
**Travel time to hospital**				
<10 minutes	1.00	1.00	1.00	1.00
10–30 minutes	0.55 (0.3–0.9)	0.93 (0.33–2.34)	1.7 (0.84–3.19)	2.88 (0.99–7.69)
30–1 hour	0.51 (0.24–0.96)	0.81 (0.28–2.09)	1.16 (0.53–2.3)	1.68 (0.58–4.44)
>1 hour	0.47 (0.19–1.01)	1.2 (0.39–3.2)	1.22 (0.37–3.32)	3.58 (1.06–10.6)
**Random effects**				
Month of admission (Seasonal variance parameter)	0.01 (0–0.06)	0.08 (0.01–0.31)	0.01 (0–0.08)	0.46 (0.08–1.56)
Age (Variance parameter)	0 (0–0)	0 (0–0)	0 (0–0)	0 (0–0)
Spatial variance	0.17 (0.01–0.9)	0.37 (0.12–0.92)	0.54 (0.06–2.06)	0.08 (0–1.22)
Spatial range (Decimal degree)	0.03 (0.01–0.15)	0.23 (0.09–0.54)	0.11 (0.03–0.38)	0 (0–0.02)

**Figure 2:**
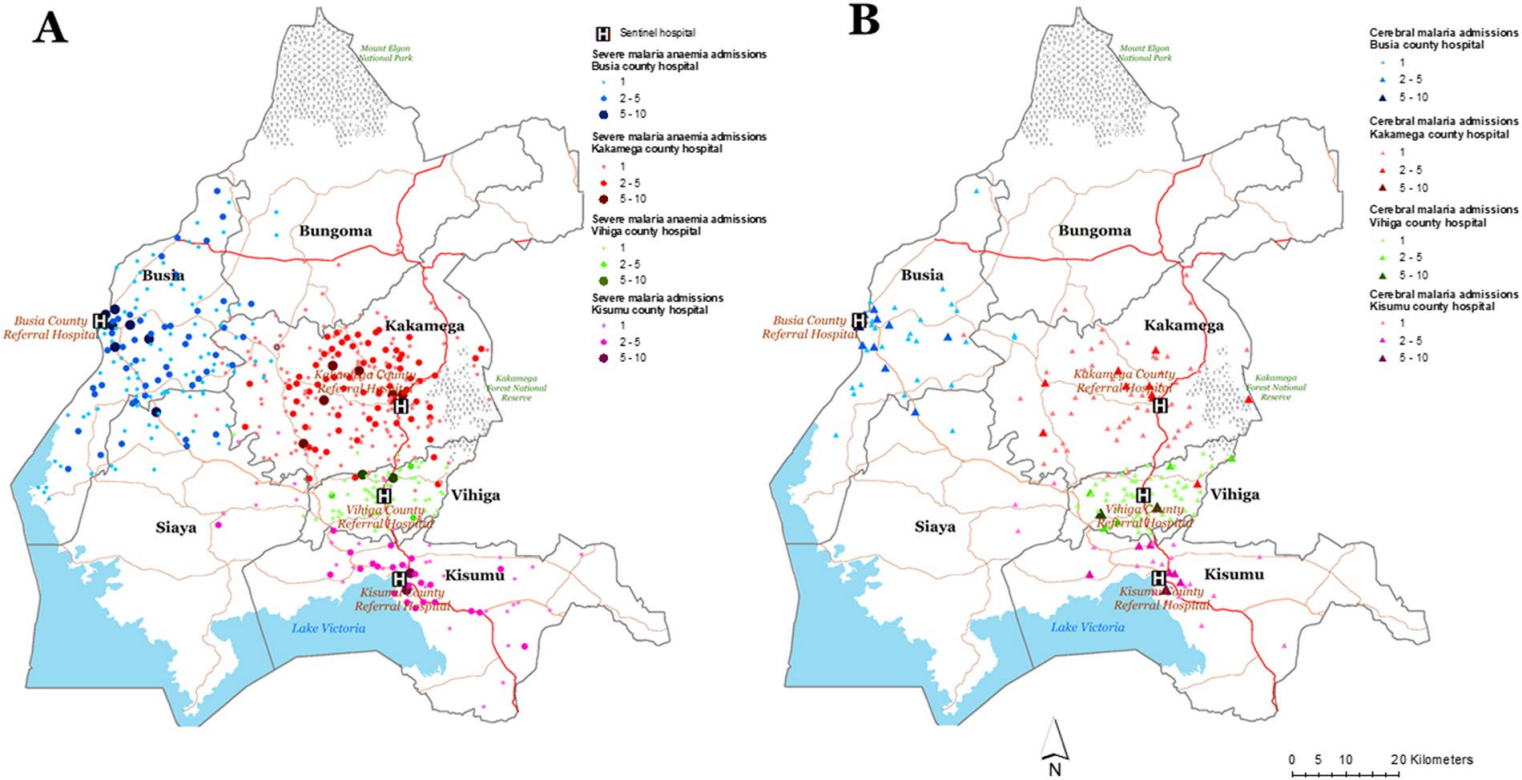
Distribution of severe malaria anaemia (SMA) (n = 1001) and cerebral malaria (CM) (n = 272) admissions at the four sentinel hospitals.

Figure 2 shows the spatial variation of CM and SMA admission at the four sentinel hospitals. Form individual-level analysis, CM and SMA admission rates varied by travel-time from the sentinel hospital. For example, at Busia county referral hospital, the rate of CM admissions was lower with increasing travel-time to the hospital (0.67 (0.36–1.16) at 30 minute and 0.17 (0.06–0.43) at >1 hour) but there was little variation in the rates admissions for SMA admissions by travel time (0.55 (0.30–0.90) at 30 minutes and 0.47 (0.19–1.01) at >1 hour). For the other three hospitals, an opposite trend of higher rates of CM and SMA admissions with increasing travel-time from the hospital was observed. The effect of distance was less marked for the severe malaria admissions at these three hospitals. For example, at Kakamega county and teaching referral hospital, the rates of admission for CM were 0.81 (0.22–3.00) at 30 minutes and 2.13 (0.6–7.95) at >1 hour compared to 0.93 (0.33–2.34) and 1.20 (0.39–3.20) for SMA, respectively).

The rate of admission was also lower outside of predicted hospital catchment compared to rates inside the catchment. For example, at Busia hospital, CM admission was lower by 69% outside the predicted catchment (posterior median relative rate 0.39 95% Bayesian Credible Interval 0.18–0.75) (Table 1) and by 66% for SMA admissions 0.34 (0.18–0.55). The rate of admission outside the catchment were also lower for Vihiga (0.59 (0.24–1.27) and 0.53 (0.25–1.00) for CM and SMA admissions outside the catchment, respectively.

## Discussion

This study provides an example of how in-patient malaria admission records can be used in a mathematical-statistical framework to delineate catchments. Findings suggest a variation in geographic catchment representative of malaria cases reaching level 4 or level 5 hospital. The largest geographic catchment size was at Kakamega county teaching and referral hospital at 322.0 km^2^ and smallest at 24 km^2^ at Vihiga county hospital. For severe malaria, there were little differences in SMA and CM admissions by distance or travel time. The estimated travel time considering the four sentinel hospitals was (45.90 minutes, IQR 22.41–70.99 minutes) for SMA and (38.5 minutes, IQR 20.51–55.76 minutes) for CM. Admission rates for SMA and CM were highest before 30 minutes or after 1-hour travel time (Table 3) and there was a lower likelihood of presentation outside of the predicted hospital catchment.

There were very little differences in travel time or distance between SMA and CM children reaching the hospital. For this group admission was also more likely for communities <1 hour from the hospital. Given a high density of health facilities in the study area, it is likely that presentation at a greater travel time to the hospital is attenuated by competition from other health facilities capable of providing in-patient paediatric services. Elsewhere, travel time is a documented risk factor for child mortality related to malaria^18,20,27^. Moreover, when the density of health facilities is low, previous research has shown a decreasing presentation at the hospital with increasing distance (distance-decay)^18,20,28^. In contrast, when the density of health facilities was higher studies have shown a less marked effect of distance^29,30^. Therefore, for this region, the geographic distance may not be the only barrier for hospital care. A variation in care-seeking practices for malaria may contribute to a delayed presentation at the hospital^31–33^. As observed in previous studies, these may include but not limited to: care-givers recognition of danger signs (symptoms), household decision-making, mother’s education level, and the use of informal care or self-medication^34–37^.

These community-level factors combined with health system-level factors contribute to estimated variability in catchments although these are representative of cases reaching the hospital. Larger hospitals such as Kakamega referral and teaching hospital may be more attractive in the presence of danger signs that overcomes distance barriers contributed by referral system for severe malaria cases from lower-level facilities. In Uganda, the rate of referral completion, recommended by community health workers, was less than 50% but 2.8 times higher amongst children with danger signs^38^. Our study did not record information on referral malaria cases or factors associated with referrals. However, Fig. 1B depicts communities around the hospital that might require improved transportation for severe malaria conditions requiring emergency care.

The novelty introduced by the two-component approach allowed for zero inflation by including EAs with zero observations. The Bayesian modelling approach quantified spatial dependencies in observed malaria admissions^39^ and incorporated measures of uncertainty^40,41^. The spatial-statistical approach had advantages of examining the distance at which the spatial autocorrelation of observations is minimised^42^. The approach can be improved by including other parameters of health care access based on data from community surveys in a generalized spatial regression framework. For example, the inclusion of socio-demographic factors impacting malaria care-seeking at the community level. From a hospital perspective, the method could be extended by explicitly modelling a competition parameter combining aspects of population demand for malaria treatment with hospital availability. Such an undertaking would, however, require a definition of competing hospitals with geocoded admission data, and a knowledge of care-seeking behaviour for malaria at community level.

The findings presented should be interpreted with reference to limitations of available data both for the numerator and denominators. Inaccuracies in the estimation of census population (the denominator), or, by assuming a static EA population may contribute to less precision in estimation. A constant county-level inter-census growth rate was used to project EA census counts while short-term changes in population structure considered at the EA are unknown. For the numerator, a variation in malaria treatment-seeking behaviour contributes to incomplete case registry at the hospital. This study used malaria admissions, it is likely that some malaria cases at the community do not reach the hospital. Thus, the catchment is representative of malaria cases reaching hospital rather than overall healthcare-seeking for all health conditions in the population. The methodology could be improved by geocoding all hospital-related admissions beyond the scope of the current study. In general, an in-depth understanding of treatment-seeking would require additional community surveys. However, for rare conditions requiring emergency care, household-level surveys have both sampling and ethical challenges. Our analysis of travel time followed previous frameworks^43–45^ by assuming one mode of travel around major roads (motorized) or walking across other landscapes. In reality, combined modes of travel may be used by an individual with a variation at a household level confounded by social-economic factors^46^. Due to the scope of the study such fine-scale variations were not examined along with maternal characteristics.

The delineation of disease-specific hospital catchments is important in identifying populations marginalized from health services^19,47^ and these are best defined with data-driven approaches that include demographic characteristics of populations and clinical case observations. In general, if the catchment population is identified, then various population-level disease indicators could be monitored^48–50^. For emergency care, catchments may be useful for designing improved access to ambulatory or referral care, for example, in targeting interventions such as rectal artesunate in patients with CM^51^. In addition, hospital catchment representative of malaria care-seeking could also inform health system impact evaluation cluster randomised controlled interventions such as the RTS,S/AS01 malaria vaccine pilot implementation^52,53^. In such vaccine impact evaluation studies, an imbalanced selection of catchment communities could affect estimates of effectiveness^54^. Other previous routine surveillance studies include the evaluation of the MenAfriVac vaccine for meningitis^55^, and the Rotavirus vaccine^56^.

This study presented a detailed spatial-statistical analysis using malaria admission to delineate catchments and conducted a spatial analysis of SMA and CM. While our methods controlled for spatial autocorrelation, we found that travel time to the hospital was similar between SMA and CM. Additionally, the rate of admissions was lower outside the predicted hospital catchment areas. While this study focused on retrospective events over a 2-year period, an important aspect that should be considered for future studies is an empirical understanding of how, when and what choices caregivers make when seeking care for CM and SMA.

## Methods

### Study region and hospitals

**Figure 3:**
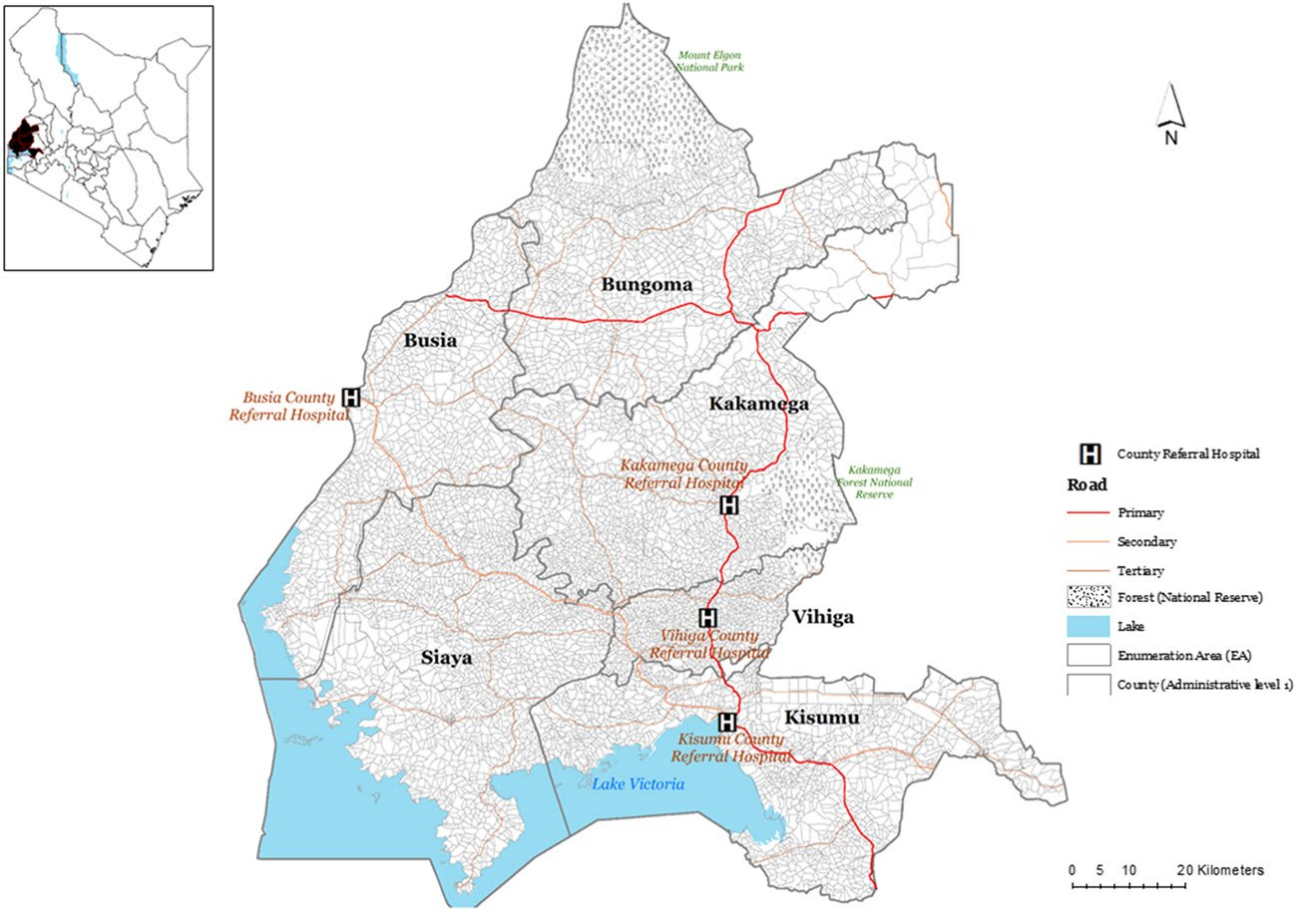
Map showing the six counties (Busia, Kakamega, Vihiga, Kisumu, Siaya and Bungoma) and the four sentinel hospitals from which in-patient paediatric malaria admissions data was assembled. The four study hospitals were Busia county referral level-4 hospital with 185 beds, Kakamega provincial general level 5 hospital with 449 beds, Vihiga county level 4 hospital with 195 beds and the Vihiga county referral level 4 hospital with 160 beds. The red lines show main primary (trunk roads), secondary (minor trunk, single carriage roads) and tertiary roads (single carriage roads that connect truck roads) produced (via mapping) by the ministry of transport, infrastructure housing, urban development and public works. The small polygons within the county boundaries represent census Enumeration Areas (EAs, *n* = 7520) based on the 2009 housing census from the Kenya National Bureau of Statistics (KNBS). An EA is a small polygon (a village) estimated to contain 100 households during national household and population census for 2009.

The study was conducted in stable, endemic malaria setting^26^ in Western Kenya (Fig. 3). The region has a high population density of approximately 600 people per square kilometer (KNBS, 2010). Enhanced routine hospital paediatric surveillance has been in operation since 2013 using training, systematic symptom and laboratory result documentation, and electronic data capture tools. Further details of this Clinical Investigation Network (CIN) are provided elsewhere^57–59^. Data were recently re-analyzed to define the clinical characteristics of malaria admissions over two complete years, either side of a national doctor’s strike, December 2015 to November 2016 and November 2017 to October 2018 from four county referral hospitals (Busia, Kakamega (teaching and referral), Vihiga, and Kisumu; Fig. 3)^60^. Each hospital in Fig. 3 is a major level 4 and level 5 hospital serving as a link between the national referral hospitals and the sub-county-level health facilities^61^. In addition to routine service delivery, the level4/5 hospitals provide a range of services including but not limited to paediatric, surgical services (have surgical ward), medical ward, education outreach, pharmaceutical, laboratory, accident and emergency unit, medical imaging, blood donation units, emergency obstetric care and anesthesiology.

### Hospital malaria admissions

The database comprised children of 1 month of age up to 14 years, where a malaria diagnosis was established by a clinician based on laboratory confirmation (positive smear) and clinical observations including history, clinical signs (presence of fever, temperature >37.5 °C)^60^. Neurological status was based on observations of whether the child was alert, or able to respond to voice or pain or was unconscious, (AVPU) scale. Cerebral malaria (CM) was defined among children who were unconscious (U) or could only respond to a painful stimulus (P)^60^. This combined clinical score approximates to a Blantyre Coma Score of <4. Severe malarial anaemia (SMA) was defined based on a combination of admission haemoglobin (Hb) < 5 g/dl, or using clinical signs of severe pallor or where blood transfusion was prescribed where haemoglobin measurement was missing^60^.

### Address matching malaria admissions to enumeration areas (EAs)

Each child’s electronic data record did not include information on the village of residence. However, details of the child’s residence were written on the medical records. For each malaria admission, the in-patient medical record was retrieved at each hospital’s records office. Information on place of residence was retrieved including county, sub-country, the nearest school, the nearest health facility or market centre. The village of residence was linked to the 2009 census enumeration areas (EAs) obtained from the Kenya National Bureau of Statistics (KNBS) (Fig. 3) and checked in ArcGIS 10.5 ESRI Geographic Information Systems (GIS) software for errors or anomalies. An EA in the 2009 census comprised of approximately 100 households that could be enumerated in a single census night and was on average 1.5 km^2^. The inter-census population growth rates and population age structure (5-year intervals) was obtained from county-level summaries provided by the KNBS^62^. The population growth rate was used to project the counts at EA-level to the study period (2017–2018) while the average proportion of 0–14 years at the county-level was used to correct for age (0–14 years). Maps on the spatial distribution of admission were produced at the EA-level converted to cluster points based on the EA centroid using ESRI ArcGIS software.

### Ancillary spatial data

Ancillary geographic layers for roads, elevation and land cover were assembled. Roads comprised of primary (trunk roads), secondary (minor trunk, single carriage roads), and tertiary roads (single carriage roads that connect trunk roads) produced by the Ministry of Transport, Infrastructure, Housing, Urban Development and Public Works via national mapping in 2007. Land cover map for the region was extracted from the 2009 MERIS GlobCover version 2.3 that has 22 land cover classes defined based on the United Nations (UN) land cover classification system^63^. The elevation (DEM) data was obtained from HydroSHEDS dataset based primarily on NASA’s Shuttle Radar Topography Mission (SRTM)^64^.

### Estimation of spatial distance and travel time to hospital

Euclidean distance from the centroid of the EA to hospital and to the nearest road was computed from ArcGIS ESRI 10.5 using the road data set. Distance to the nearest road was used in the predictive catchment modelling described in the next section. For travel time, a spatial analysis was conducted using AccessMod version 5 software^65^ based on land cover, elevation and roads^18,43,45,66^. In brief, a new raster land cover layer was generated by combining the Globcover layer with roads. Travel time to the main hospital was analysed by specifying travel speed (impedance) to various land cover classes. For example, a major highway was assigned a speed of 80 kilometres per hour (80 km/h) in line with national road regulations assuming motorised travel. For the other all-weather footpaths, a 4 km/h walking correction was used^65^. The algorithm for deriving travel time in each land cover class included a slope correction derived from elevation data with travel speeds calculated for each degree rise of slope based on Tobler’s equation $$(V={6}^{\ast }\,\exp (\,-\,3.5abs[Tan(slope\,in\,\begin{array}{c}\deg rees/57.3\end{array}+0.05)]))$$^67^ where *V* is the calculated speed.

### Novel statistical methodology for delineating catchment

The use of admission rates to characterise catchment areas was first described in 1956^68^, adapted during the 1970s^69^ and mathematically defined in early 1980s^70^. Here, a spatial-statistical approach was undertaken. It was assumed that the likelihood of admission varied spatially. Therefore, all EAs in the region encompassing geo-referenced admissions for each hospital were used in the prediction of catchment areas.

Let $N({S}_{i})$ denote the EA population for the number of children under 15 years of age at risk of malaria. A Bayesian hierarchical zero-inflated Poisson regression was used with probability mass function given as^71,72^,

1 $$f({y}_{i}|\lambda ,\varphi ) \sim \left\{\begin{array}{c}{\varphi }_{i}+(1-{\varphi }_{i}){e}^{-\lambda }\\ (1-{\varphi }_{i})\frac{{e}^{-\lambda }{\lambda }^{{y}_{i}}}{{y}_{i}!}\end{array}\right.\,\begin{array}{c}{y}_{i}=0\\ {y}_{i} > 0\end{array}$$

for the $i$^th^ observation and $0 < {\phi }_{i} < 1$. Thus, the probability was defined via the two-component mixed model containing a Bernoulli zero-inflation and a general Poisson counts model ($\Pr (X=k)={\lambda }^{k}{e}^{-\lambda }/k!$). The Poisson counts model defined cases form EA seen at the hospital. Each hospital modelling was conducted separately rather than a pooled analysis due to differential qualities of the hospital e.g. size. Covariates were specified through $\lambda$ as:

2 $$\log (\lambda ({s}_{i}))=\,\log (N({s}_{i}))+{\alpha }_{0}+x{({s}_{i})}^{T}{\beta }_{1}+w({s}_{i})$$

where $\log (N({s}_{i}))$ is the offset term, ${\alpha }_{0}$ an intercept, *β* is the regression parameter for a covariate as the spatial distance from the centroid of the EA to the nearest road. $w(s)$ represents a mean-zero Gaussian Process (GP) $w(s) \sim GP\{0,C(s,s^{\prime} )\}$ associated with the spatial association between observations. The associated covariance between two pairs of locations was modelled via a Matérn covariance matrix expressed as $C(s,s^{\prime} )={\sigma }^{2}\rho (s,s^{\prime} ;\theta )$ where $\rho$ is the correlation function and $\theta$ includes parameters quantifying the rate of decay and smoothness of realization.

Computation was carried out via Gaussian Markov Random Fields (GMRF)^73^ using a stationary SPDE^42,74,75^. More details of GMRF is presented in the supplementary information. The Bayesian specification was complete by assigning non-informative priors to the fixed and SPDE parameters. For the SPDE, penalized complexity priors^76^ were used for the spatial specification thus $\theta ={(\log (\tau ),\log (k))}^{T}$, $\log \,\tau  \sim N({m}_{\tau },{l}_{\tau }^{2})$ and $\log \,k \sim N({m}_{k},{l}_{k}^{2})$ with ${m}_{k}$ as the spatial range representing approximately 1/5 of the domain area characterized by a convex hull of all admissions. Lastly, ${m}_{\tau }$ was selected to have a small standard deviation (e.g. 0.1).

### Model selection and sensitivity analysis

Model selection was based on deviance information criterion (DIC), the Watanable Akaike Information criterion (WAIC)^77^, cross-validated mean logarithmic score (MLS). Additionally, the root mean square error (RMSE), the percentage of variance explained by the model were calculated from a 30% subset of EA clusters selected randomly. The cross-validation approach comprised of calculating the conditional predictive ordinate (CPO), which is the probability of observing a value given all other data and was examined for all observations^78^.

### Spatial determinants of severe malaria admission

To examine the spatial determinants of CM and SMA admissions at the four hospitals, an individual-level analysis was conducted with dichotomy ${y}_{i}=1$ representing a child that had CM or SMA. A Bayesian hierarchical logistic regression model for CM and SMA with a spatial effect of the following form was implemented,

3 $$\log \,it(p({s}_{i}))={\alpha }_{0}+x{({s}_{i})}^{T}{\beta }_{1}+w({s}_{i})$$

where $w(s)$ was modelled as previously stated in Eq. 2 and the set of random and fixed dichotomous regression parameters. These included if CM or SMA was within the predicted catchment (categorical variable), a continuous random effect of child age rather than as a categorical variable, a random effect on the month of admission, and an estimated travel-time to paediatric hospital (<10 minutes, 10–30 minutes, 30 minutes - 1 hour, and >1 hour). Random effects allowed an examination of how the variable mean change in a continuous form. Bayesian specification was completed by assigning relevant prior information. Flat priors were assigned to the fixed effects with only the age of child and month of admission allowed to vary randomly based on a second-order random walk for flexible smoothing^79^. The precision parameters of random walk for age were assigned prior distributions using penalized complexity priors with approximate mean and variance (for age parameter (1, 0.001). For the month of admission, the precision parameters were assigned log gamma priors with parameters (0.1, 0.001). This approach was implemented using R-INLA^80,81^ and the schematic methodological framework is included in the supplementary material (Supplementary Fig. S1).

### Ethical approval and consent to participate

This work is published with the permission of the Director of KEMRI. KEMRI Scientific and Ethical Review Committee approved the CIN study (SERU number 2465 and number 3459). All malaria routine data for the study period were retrieved retrospectively in 2019 from the health records office under CIN study protocol. There was no contact made with individuals at the hospital level. Details of study regulations and can be obtained by contacting IRB https://www.kemri.org/.

## Supplementary information

Supporting Information.

## Data Availability

Data for this study are under the primary jurisdiction of the Ministry of Health in Kenya. Enquiries about using the data can be made to the KEMRI-Wellcome Trust Research Programme Data Governance Committee.

## References

[1] World Health Organization. World Malaria Report 2018. (World Health organization, Geneva, 2018).

[2] Snow RW (2017). The prevalence of Plasmodium falciparum in sub-Saharan Africa since 1900. Nat..

[3] Greenwood B, Marsh K, Snow R (1991). Why do some African children develop severe malaria?. Parasitol. Today.

[4] Marsh K (1995). Indicators of life-threatening malaria in African children. N. Engl. J. Med..

[5] World Health Organization (2014). Severe Malaria. Tropical Med. Int. Health.

[6] Sypniewska P (2017). Clinical and laboratory predictors of death in African children with features of severe malaria: a systematic review and meta-analysis. BMC Med..

[7] Gwer S (2012). Changing trends in incidence and aetiology of childhood acute non-traumatic coma over a period of changing malaria transmission in rural coastal Kenya: a retrospective analysis. BMJ Open..

[8] Maitland K (2015). Management of severe paediatric malaria in resource-limited settings. BMC Med..

[9] Kiguli S (2015). Anaemia and blood transfusion in African children presenting to hospital with severe febrile illness. BMC Med..

[10] Maitland K (2019). Immediate Transfusion in African Children with Uncomplicated Severe Anemia. N. Engl. J. Med..

[11] Varo R (2018). Adjunctive therapy for severe malaria: a review and critical appraisal. Malar. J..

[12] Reynolds, T. A. *et al*. in *Disease Control Priorities: Improving Health and* Reducing *Poverty* (eds rd *et al*.) (The World Bank, 2017). 30212058

[13] Moresky RT (2019). Advancing research on emergency care systems in low-income and middle-income countries: ensuring high-quality care delivery systems. BMJ Glob. Health.

[14] Razzak J, Beecroft B, Brown J, Hargarten S, Anand N (2019). Emergency care research as a global health priority: key scientific opportunities and challenges. BMJ Glob. Health.

[15] Zinszer K (2014). Determining health-care facility catchment areas in Uganda using data on malaria-related visits. Bull. World Health Organ..

[16] Jones S, Wardlaw J, Crouch S, Carolan M (2011). Modelling catchment areas for secondary care providers: a case study. Health Care Manag. Sci..

[17] Guagliardo MF (2004). Spatial accessibility of primary care: concepts, methods and challenges. Int. J. Health Geogr..

[18] Manongi R (2014). Inpatient child mortality by travel time to hospital in a rural area of Tanzania. Trop. Med. Int. Health.

[19] Schellenberg JA (1998). An analysis of the geographical distribution of severe malaria in children in Kilifi District, Kenya. Int. J. Epidemiol..

[20] Schoeps A, Gabrysch S, Niamba L, Sie A, Becher H (2011). The effect of distance to health-care facilities on childhood mortality in rural Burkina Faso. Am. J. Epidemiol..

[21] Moisi JC (2011). Sensitivity of hospital-based surveillance for severe disease: a geographic information system analysis of access to care in Kilifi district, Kenya. Bull. World Health Organ..

[22] Cromley, E. K. & McLafferty, S. L. *GIS and public health*. (Guilford Press, 2002).

[23] O’Meara WP (2014). Heterogeneity in health seeking behaviour for treatment, prevention and urgent care in four districts in western Kenya. Public. Health.

[24] Were V (2018). Socioeconomic health inequality in malaria indicators in rural western Kenya: evidence from a household malaria survey on burden and care-seeking behaviour. Malar. J..

[25] National Malaria Control Programme - NMCP/Kenya, Kenya National Bureau of Statistics - KNBS & ICF International. Kenya Malaria Indicator Survey 2015. (NMCP, KNBS, and ICF International, Nairobi, Kenya, 2016).

[26] Macharia PM (2018). Spatio-temporal analysis of Plasmodium falciparum prevalence to understand the past and chart the future of malaria control in Kenya. Malar. J..

[27] Kazembe LN, Kleinschmidt I, Sharp BL (2006). Patterns of malaria-related hospital admissions and mortality among Malawian children: an example of spatial modelling of hospital register data. Malar. J..

[28] Ippolito MM (2018). Risk Factors for Mortality in Children Hospitalized with Severe Malaria in Northern Zambia: A Retrospective Case-Control Study. Am. J. tropical Med. Hyg..

[29] Moïsi JC (2010). Geographic access to care is not a determinant of child mortality in a rural Kenyan setting with high health facility density. BMC Public. Health.

[30] Rutherford ME (2009). Access to health care and mortality of children under 5 years of age in the Gambia: a case-control study. Bull. World Health Organ..

[31] Sundararajan R (2015). Sociocultural and structural factors contributing to delays in treatment for children with severe malaria: a qualitative study in southwestern Uganda. Am. J. tropical Med. Hyg..

[32] Kassile T, Lokina R, Mujinja P, Mmbando BP (2014). Determinants of delay in care seeking among children under five with fever in Dodoma region, central Tanzania: a cross-sectional study. Malar. J..

[33] Wasunna B (2015). The Impact of a Community Awareness Strategy on Caregiver Treatment Seeking Behaviour and Use of Artemether-Lumefantrine for Febrile Children in Rural Kenya. PLoS ONE.

[34] Bigogo G (2010). Health-seeking patterns among participants of population-based morbidity surveillance in rural western Kenya: implications for calculating disease rates. Int. J. Infect. Dis..

[35] Ilunga-Ilunga F, Leveque A, Ngongo LO, Laokri S, Dramaix M (2015). Treatment-seeking Paths in the Management of Severe Malaria in Children under 15 Years of Age Treated in Reference Hospitals of Kinshasa, Democratic Republic of Congo. Trop. Med. Health.

[36] Kagabo DM (2018). Care-seeking patterns among families that experienced under-five child mortality in rural Rwanda. PLOS ONE.

[37] Kallander K (2008). Delayed care seeking for fatal pneumonia in children aged under five years in Uganda: a case-series study. Bull. World Health Organ..

[38] Nanyonjo A (2015). Estimating the cost of referral and willingness to pay for referral to higher-level health facilities: a case series study from an integrated community case management programme in Uganda. BMC Health Serv. Res..

[39] Banerjee S, Gelfand AE, Polasek W (2000). Geostatistical modelling for spatial interaction data with application to postal service performance. J. Stat. Plan. Inference.

[40] Diggle, P. J. & Ribeiro, P. J. *Model-based geostatistics*. (Springer, 2007).

[41] Diggle PJ, Menezes R, Su T-l (2010). Geostatistical inference under preferential sampling. J. R. Stat. Society: Ser. C..

[42] Fuglstad Geir-Arne, Simpson Daniel, Lindgren Finn, Rue Håvard (2015). Does non-stationary spatial data always require non-stationary random fields?. Spatial Statistics.

[43] Alegana V (2012). Spatial modelling of healthcare utilisation for treatment of fever in Namibia. Int. J. Health Geographics.

[44] Ouma PO (2018). Access to emergency hospital care provided by the public sector in sub-Saharan Africa in 2015: a geocoded inventory and spatial analysis. Lancet Glob. Health.

[45] Macharia PM, Odera PA, Snow RW, Noor AM (2017). Spatial models for the rational allocation of routinely distributed bed nets to public health facilities in Western Kenya. Malar. J..

[46] Mpimbaza A, Ndeezi G, Katahoire A, Rosenthal PJ, Karamagi C (2017). Demographic, Socioeconomic, and Geographic Factors Leading to Severe Malaria and Delayed Care Seeking in Ugandan Children: A Case-Control Study. Am. J. Trop. Med. Hyg..

[47] Gilmour SJ (2010). Identification of Hospital Catchment Areas Using Clustering: An Example from the NHS. Health Serv. Res..

[48] Alegana VA (2013). Estimation of malaria incidence in northern Namibia in 2009 using Bayesian conditional-autoregressive spatial-temporal models. Spat. Spatio-temporal Epidemiol..

[49] Boyce RM (2016). Practical Implications of the Non-Linear Relationship between the Test Positivity Rate and Malaria Incidence. PLOS ONE.

[50] Oduro AR (2011). Health Centre Surveys as a Potential Tool for Monitoring Malaria Epidemiology by Area and over Time. PLoS One.

[51] Gomes MF (2009). Pre-referral rectal artesunate to prevent death and disability in severe malaria: a placebo-controlled trial. Lancet.

[52] World Health organization. Malaria Vaccine Rainbow Tables., http://www.who.int/vaccine_research/links/Rainbow/en/index.html (2017).

[53] Schwartz L, Brown GV, Genton B, Moorthy VS (2012). A review of malaria vaccine clinical projects based on the WHO rainbow table. Malar. J..

[54] Ivers NM (2012). Allocation techniques for balance at baseline in cluster randomized trials: a methodological review. Trials.

[55] Trotter CL (2017). Impact of MenAfriVac in nine countries of the African meningitis belt, 2010-15: an analysis of surveillance data. Lancet Infect. Dis..

[56] Omore R (2019). Rates of hospitalization and death for all-cause and rotavirus acute gastroenteritis before rotavirus vaccine introduction in Kenya, 2010-2013. BMC Infect. Dis..

[57] Ayieko P (2016). Characteristics of admissions and variations in the use of basic investigations, treatments and outcomes in Kenyan hospitals within a new Clinical Information Network. Arch. Dis. Child..

[58] Irimu G (2018). Approaching quality improvement at scale: a learning health system approach in Kenya. Arch. Dis. Child..

[59] Tuti T (2016). Innovating to enhance clinical data management using non-commercial and open source solutions across a multi-center network supporting inpatient pediatric care and research in Kenya. J. Am. Med. Inform. Association: JAMIA.

[60] Akech, S. *et al*. The clinical profile of severe paediatric malaria in an area targeted for routine RTS,S/AS01 malaria vaccination in Western Kenya. *Clinical Infectious Diseases*, 10.1093/cid/ciz844 (2019). 10.1093/cid/ciz844 PMC7353324 31504308

[61] Ministry of Health. Kenya Master Health Facility List, http://kmhfl.health.go.ke/#/home (2019).

[62] Kenya National Bureau of Statistics. The 2009 Kenya population and housing census. (KNBS, Nairobi, 2010).

[63] Arino, O. *et al*. In *Proceedings of the International Geoscience and Remote Sensing Symposium (IGARSS)* 2007 2412 - 2415 (IEEE International, 2007).

[64] Lehner B, Verdin K, Jarvis A (2008). New Global Hydrography Derived From Spaceborne Elevation Data. Eos, Trans. Am. Geophys. Union..

[65] Ray N, Ebener S (2008). AccessMod 3.0: computing geographic coverage and accessibility to health care services using anisotropic movement of patients. Int. J. Health Geographics.

[66] Bennett A (2014). A methodological framework for the improved use of routine health system data to evaluate national malaria control programs: evidence from Zambia. Popul. Health Metr..

[67] Tobler, W. Three presentations on geographical analysis and modeling: National Center for Geographic Information and Analysis. (University of California, Santa Barbara, Santa Barbara, CA93106-4060, 1993).

[68] Norman TJB (1956). Statistics in Hospital Planning and Design. J. R. Stat. Society. Ser. C..

[69] Bailey, N. T. J. Mathematics, statistics, and systems for health. (John Wiley and Sons Ltd., Baffins Lane, Chichester, Sussex, 1977).

[70] Senn SJ, Samson WB (1982). Estimating Hospital Catchment Populations. J. R. Stat. Society. Ser. D..

[71] Ghosh SK, Mukhopadhyay P, Lu J-C (2006). Bayesian analysis of zero-inflated regression models. J. Stat. Plan. Inference.

[72] Lambert D (1992). Zero-inflated Poisson regression, with an application to defects in manufacturing. Technometrics.

[73] Rue, H. & Held, L. Gaussian Markov Random Fields: Theory and Applications (Chapman & Hall/CRC Monographs on Statistics & Applied Probability). (Chapman and Hall/CRC, 2005).

[74] Lindgren, F. & Rue, H. Bayesian Spatial and Spatio-temporal Modelling with R-INLA. (Norwegian University of Science and Technology, Norway, Trondheim, 2013).

[75] Lindgren F (2013). Continuous domain spatial models in R-INLA. ISBA Bull..

[76] Fuglstad Geir-Arne, Simpson Daniel, Lindgren Finn, Rue Håvard (2018). Constructing Priors that Penalize the Complexity of Gaussian Random Fields. Journal of the American Statistical Association.

[77] Watanabe S (2010). Asymptotic Equivalence of Bayes Cross Validation and Widely Applicable Information Criterion in Singular Learning Theory. J. Mach. Learn. Res..

[78] Held, L., Schrödle, B. & Rue, H. In *Statistical Modelling and Regression Structures* (eds G Tutz & T Kneib) (Heidelberg, 2009).

[79] Lindgren F, Rue H (2008). On the Second-Order Random Walk Model for Irregular Locations. Scand. J. Stat..

[80] Rue H, Martino S, Chopin N (2009). Approximate Bayesian inference for latent Gaussian models by using integrated nested Laplace approximations. J. R. Stat. Society: Ser. B.

[81] Lindgren F, Rue H, Lindström J (2011). An explicit link between Gaussian fields and Gaussian Markov random fields: the stochastic partial differential equation approach. J. R. Stat. Society: Ser. B.

