# Supplementary material for: Estimating hospital catchments from in-patient admission records: a spatial statistical approach applied to malaria
Source: Sci Rep. 2020 Jan 28;10:1324. doi: 10.1038/s41598-020-58284-0 (PMC6987150; doi:10.1038/s41598-020-58284-0)
Supplement: Supplementary file 1 — Supporting Information. [file 41598_2020_58284_MOESM1_ESM.pdf]

Supplementary Information

A Bayesian approach for investigating hospital access and catchment platforms to test malaria vaccine efficacy

Victor A Alegana[1,2,3*], Cynthia Khazenzi[1], Samuel O Akech[1], Robert W Snow[1,4]

Corresponding author: Alegana Victor; email: valegana@kemri-wellcome.org

Table of Contents

Overview of methodology 3
A hierarchical model for hospital catchments 3
Gaussian Markov Random Field (GMRF) 3
Model scoring rules 5
Additional results 6
Sensitivity and validation for catchment model 6
Predictive catchment areas for malaria admissions 7
References 8

Overview of methodology

The Enumeration Areas (EAs) were plotted in a geographic information system (GIS) and converted to point locations for geostatistical analysis. The prediction of catchment areas was based on malaria admissions excluding severe malaria.

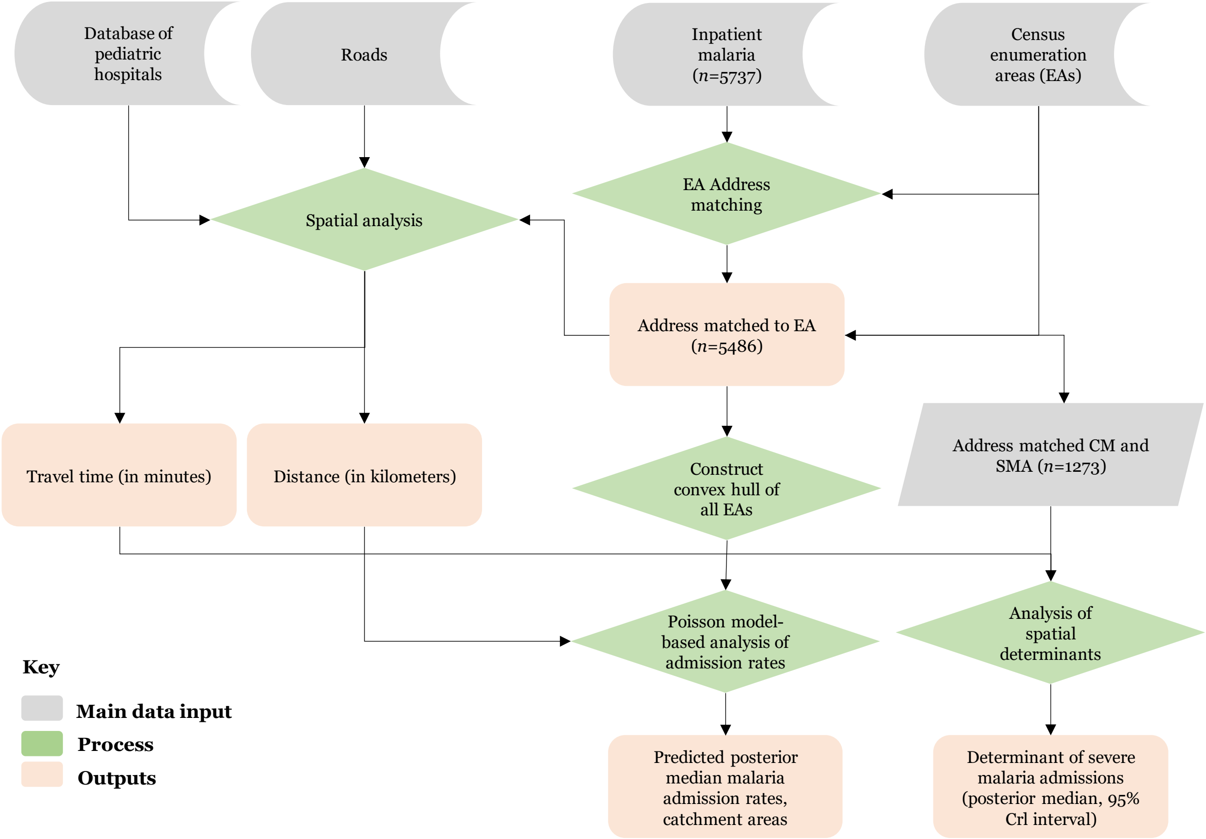

Figure S1: Schematic overview of the methodology for analysis malria admission rates and catchments areas, and characteristics associated with cerebral malaria in four hospitals in Western Kenya.

A hierarchical model for hospital catchments

Gaussian Markov Random Field (GMRF)

GMRFs are widely used in Bayesian hierarchical models applications due to their relative ease of implementation and Markovian properties [1]. In geostatistics each observation is a Random Variable (RV) is a drawn from a distribution (usually Gaussian). Realisations (outcomes) are then drawn from a random function (RF). Thus, the RF at a location in space can have a series of outcomes (realizations) and relate to another point at a different location based on a

function of distance (generally Euclidean distance) [2,3]. A random vector $x = (x_1,.......x_n)^T$ can be defined as a GMRF with mean μ and positive definite precision matrix Q with density as:

$$\pi(x) = 2\pi^{-n/2} \, |Q|^{1/2} \exp[-\frac{1}{2}(x-\mu)^T Q(x-\mu)]$$

where π is the density and Q is the precision matrix with covariance matrix $\Sigma = Q^{-1}$. Two RV x_i and x_j $(i \neq j)$ are conditionally independent with conditional density $\pi(.|.)$ if $\pi(x_i\, x_j \,|\, x_{-ij}) = \pi(x_i \,|\, x_{-ij}) \cdot \pi(x_j \,|\, x_{-ij})$. For a GMRF, Q is usually sparse allowing for fast computation based on Cholesky decomposition in Integrated Nested Laplace Approximation (INLA) see [1]. The joint posterior distribution $\pi(y\,|\,x,\theta)$, where y are the observations, x latent Gaussian variable with θ hyperparameters can be written as:

$$\pi(x, y\,|\,\theta) \;\; \alpha \;\; \pi(\theta)\pi(x\,|\,\theta)\prod \pi(y_i\,|\,x_i, \theta)$$

$$\alpha \;\; \pi(\theta) \;\; |Q|^{1/2} \exp[-\frac{1}{2}x^T \;\; Q(\theta) \;\; x + \sum \; \log\{\pi(y_i\,|\,x_i, \theta\}$$

For Bayesian inference the desired marginal distribution is

$$\pi(x_i\,|\,y_i) \;\; = \;\; \int \pi(x_i\,|\,\theta, y)\pi(\theta\,|\,y)\,d\theta$$

$$\pi(\theta_j\,|\,y) \;\; = \;\; \int \pi(\theta\,|\,y) \;\; d\theta_{-j}$$

The INLA method approximates the curvature of the mode evaluated at suitable sampling points [4]. The difference between INLA and widely used MCMC [5] relates to computational efficiency that the former enjoys. INLA however applies for a class of Latent Gaussian Models (LGM) such as spatial models used in this study.

$$\tilde{\pi}(x_i\,|\,y_i) \;\; = \;\; \int \tilde{\pi}(x_i\,|\,\theta, y)\tilde{\pi}(\theta\,|\,y)\,d\theta$$

$$\tilde{\pi}(\theta_j\,|\,y) \;\; = \;\; \int \tilde{\pi}(\theta\,|\,y) \;\; d\theta_{-j}$$

The integral is then evaluated via a finite sum

$$\tilde{\pi}(x_i\,|\,y) \;\; = \;\; \sum_k \tilde{\pi}(x_i\,|\,\theta_k, y) \;\; \tilde{\pi}(\theta_k\,|\,y) \;\; \Delta_k$$

With Δ_k as weights at appropriate values θ_k. The approximation of $\pi(\theta | y)$ is obtained by integrating θ_{-j} from $\tilde{\pi}(\theta_j | y)$ [6,7]. The initial stage involve finding the mode at supporting points followed by a laplace approximation of $\tilde{\pi}(x_i | \theta, y)$ to the conditional marginal distribution to $\pi(x_i | \theta, y)$

$$\tilde{\pi}(\theta | y) = \left. \frac{\pi(x, \theta, \; y)}{\tilde{\pi}G(x | \theta, y)} \right|_{x \, = \, x^*(\theta)}$$

where $\tilde{\pi}G(x | \theta, y)$ is the Gaussian approximation to conditional of x evaluated at the mode $x^*(\theta)$ obtained by optimization algorithm using quasi-Newton approach [8] and

$$\tilde{\pi}(x_i | \theta, y) = \left. \frac{\pi(x, \theta, \; y)}{\tilde{\pi}GG(x_{-i} | x_i, \theta, y)} \right|_{x_{-i} = \, x^*_{-i}(x_i, \theta)}$$

Model scoring rules

There are different methods to evaluate model goodness of fit. One approach is to score based on the probabilistic values from predictive distribution of the model compared to actual observations [9]. The leave one out cross validation score using the conditional predictive ordinate (CPO) is validated based on the predictive distribution [10,11]. It is the probability of observing a value given all other data. To predict a value y_p given other values, the predictive density $P(y_p | y_{-p})$ is given by:

$$P(y_p | y_{-p}) = \int \pi(y | \theta, y_{-p}) \, \pi(\theta | y_{-p}) \, d\theta_p$$

and obtained via a finite sum with weights Δ_j:

$$P(y_p | y_{-p}) \approx \sum_{1}^{j} \pi(y_p | \theta_j, y_{-p}) \, \pi(\theta_j | y_{-p}) \, \Delta_j$$

Additional results

Sensitivity and validation for catchment model

Table S1 shows model comparison via DIC, WAIC, MLS, RMSE and fraction of variance explained. The RMSE and fraction of variance explained by the model are based on 30% subset validation dataset Busia (n=86), Kakamega (n=110), Vihiga (n=291) and Kisumu (n=70).

Table S1: Bayesian model parameters showing model mean deviance ($\overline{D}$), the effective number of parameters (PD) derived from the deviance information criterion (DIC), the DIC, the Watanabe Akaike information criterion (WAIC) and the mean logarithmic score (MLS) of the sensitivity analysis conducted around choice of SPDE priors as well as the mesh.

Busia hospital	SPDE Parameters	PD	DIC	WAIC	MLS	R.M.S.E	Fraction of variance explained
	Prior 1	24.77	4394.42	4581.36	1.08	0.0016	43.96
	Prior 2	24.80	4394.49	4581.71	1.08	0.0017	39.59
	Prior 3	24.95	4392.44	4578.18	1.08	0.0016	42.32
	Prior 4	24.84	4392.66	4577.20	1.08	0.0016	43.96
	Prior 5	24.93	4393.04	4580.34	1.08	0.0016	45.13
	Prior 6	24.88	4392.69	4577.35	1.08	0.0016	45.11
	Prior 7	24.86	4392.60	4577.16	1.08	0.0015	46.70
	Mesh 1	24.84	4392.66	4577.20	1.08	0.0015	46.70
	Mesh 2	34.78	4274.63	4477.54	1.06	0.0015	49.80
	Mesh 3	34.26	4273.26	4472.25	1.06	0.0015	49.77
	Mesh 4	47.58	4191.99	4457.06	1.04	0.0015	54.00
	Mesh 5	82.27	3517.99	3832.86	0.88	0.0015	54.56
	Mesh 6	85.02	3597.62	3937.00	0.91	0.0015	55.07
	Mesh 7	84.74	3597.33	3934.62	0.91	0.0015	55.04
	Mesh 8	82.27	3517.99	3832.86	0.88	0.0015	54.56
	Mesh 9	50.31	3987.01	4208.28	0.97	0.0015	54.53
	Mesh 10	48.82	4024.92	4253.2	0.98	0.0015	54.34
Kakamega hospital	SPDE Parameters	PD	DIC	WAIC	MLS	R.M.S.E	% Explained variance
	Prior 1	70.23	5823.78	6076.69	1.10	0.0032	25.98
	Prior 2	61.53	5775.45	5998.20	1.08	0.0022	63.57
	Prior 3	59.84	5764.78	5984.78	1.08	0.0021	68.21
	Prior 4	58.31	5756.40	5973.08	1.08	0.0018	75.43
	Prior 5	62.25	5777.21	6002.62	1.09	0.0023	62.52
	Prior 6	60.05	5764.71	5985.32	1.08	0.0019	71.13
	Prior 7	58.55	5756.40	5971.56	1.08	0.0019	74.16
	Prior 8	70.45	5823.61	6075.54	1.08	0.0022	63.23
	Prior 9	59.66	5762.89	5982.14	1.08	0.0020	70.62
	Prior 10	58.53	5756.67	5973.05	1.08	0.0018	74.27
	Mesh 1	58.55	5756.40	5971.56	1.08	0.0018	75.43
	Mesh 2	47.51	6720.18	6933.69	1.25	0.0024	55.72
	Mesh 3	58.51	5756.36	5973.23	1.08	0.0018	75.62
	Mesh 4	45.01	6682.72	6869.65	1.24	0.0025	53.44
	Mesh 5	48.29	6689.31	6902.65	1.25	0.0025	53.02
	Mesh 6	58.25	5757.30	5975.10	1.08	0.0018	75.72
	Mesh 7	58.52	5757.30	5975.10	1.08	0.0018	75.72
	Mesh 8	58.52	5757.30	5975.10	1.08	0.0018	75.72
Vihiga hospital	SPDE Parameters	PD	DIC	WAIC	MLS	R.M.S.E	% Explained variance
	Prior 1	29.46	1356.19	1398.78	1.03	0.0061	49.98
	Prior 2	29.36	1356.44	1398.89	1.03	0.0061	50.00
	Prior 3	29.36	1356.44	1398.90	1.03	0.0060	50.00
	Prior 4	34.05	1348.11	1395.45	1.03	0.0060	51.74
	Prior 5	34.12	1347.90	1395.38	1.03	0.0060	51.73
	Prior 6	34.15	1347.84	1395.30	1.03	0.0060	51.77
	Prior 7	35.00	1346.16	1394.23	1.03	0.0060	52.10
	Prior 8	35.22	1346.15	1394.53	1.03	0.0060	52.09
	Prior 9	35.29	1345.99	1394.26	1.03	0.0060	52.09
	Prior 10	35.93	1345.06	1393.95	1.03	0.0060	52.30

	Prior 11	35.83	1345.19	1394.01	1.03	0.0060	52.32
	Prior 12	36.09	1344.71	1393.96	1.03	0.0060	52.33
	Mesh 1	35.93	1345.06	1393.95	1.03	0.0060	52.33
	Mesh 2	41.21	1331.25	1383.12	1.02	0.0059	53.61
	Mesh 3	40.11	1328.96	1378.95	1.02	0.0059	53.61
	Mesh 4	40.81	1323.63	1373.55	1.01	0.0026	91.01
	Mesh 5	74.26	1155.77	1201.96	0.89	0.0026	90.79
	Mesh 6	74.22	1155.85	1201.60	0.89	0.0026	90.79
Kisumu hospital	SPDE Parameters	PD	DIC	WAIC	MLS	R.M.S.E	% Explained variance
	Prior 1	34.94	3335.88	3729.26	1.04	0.0850	27.97
	Prior 4	30.12	3289.80	3603.64	1.01	0.0675	55.02
	Prior 5	30.01	3291.09	3594.43	1.01	0.0674	55.10
	Prior 6	29.98	3289.95	3575.42	1.01	0.0674	55.12
	Prior 7	29.70	3280.50	3569.12	1.01	0.0670	55.76
	Prior 8	29.86	3280.98	3552.50	1.01	0.0669	55.90
	Prior 9	30.17	3281.77	3561.22	1.01	0.0668	55.91
	Prior 10	29.84	3274.85	3538.54	1.00	0.0665	56.36
	Prior 11	29.61	3274.56	3546.92	1.00	0.0664	56.50
	Prior 12	29.77	3274.36	3532.26	1.00	0.0641	56.47
	Mesh 1	30.81	3199.32	3426.34	0.96	0.0850	28.57
	Mesh 2	22.07	2281.92	2340.57	0.71	0.0641	56.47
	Mesh 3	22.28	2283.91	2344.47	0.71	0.0664	56.52
	Mesh 4	33.61	3194.32	3493.90	0.98	0.0664	56.50
	Mesh 5	33.47	3193.93	3499.70	0.98	0.0664	56.49

Table S2: A comparison Euclidean distance and travel time to hospital for severe malaria admissions.

Hospital	Busia County Hospital	Kakamega County Hospital	Kisumu County Hospital	Vihiga County Hospital	Total
			**Cerebral Malaria**		
Median (IQR) distance to nearest roads	0.95 (0.47 - 1.53)	0.77 (0.42 - 1.22)	1.6 (0.73 - 2.44)	1.1 (0.33 - 2.17)	0.98 (0.47 - 1.96)
Median (IQR) travel times to hospital	21.49 (12.89 - 40.88)	56.1 (36.61 - 80.84)	34.48 (18.67 - 43.53)	40.69 (25.08 - 54.82)	38.5 (20.51 - 55.76)
			**Severe Malaria Anaemia**		
Median (IQR) distance to nearest roads	1 (0.52 - 1.89)	0.76 (0.39 - 1.41)	0.98 (0.46 - 2.29)	0.7 (0.33 - 1.52)	0.9 (0.43 - 1.64)
Median (IQR) travel time to hospital	37.43 (16.72 - 61.27)	63.11 (41.59 - 80.21)	26.71 (13.71 - 47.2)	35.57 (18.95 - 54.82)	45.9 (22.41 - 70.99)

Predictive catchment areas for malaria admissions

Table S2 shows the predicted spatial range based on the two-component Bayesian zero-inflated Poisson regression. The modelled spatial range from modelling was approximately 7 km for Busia paediatric malaria admissions (Posterior median 7.76, 95% Bayesian credible Interval 6.65 – 9.98) and for Kakamega hospital (7.70, 6.61-9.90) but approximately half at 3 km (3.32, 2.21 – 4.43) and 4 km (4.40, 3.33 – 6.65) for inpatient malaria admissions at Vihiga

and Kisumu hospitals, respectively. This suggested higher geographic access and use at close proximity to the hospital (e.g. within 7km in Kakamega) and thereafter a decay in usage attenuated by other competing pediatric hospitals. Thus both Busia and Kakamega had predicted larger catchment areas while on average children admitted in the other two hospitals travelled a shorter comparable distance.

Table S3: Posterior median and the 95% credible intervals for parameters of Bayesian Poisson regression for modelling hospital malaria admission rates and catchments. The spatial range parameter (in degrees' decimals) corresponds to a distance where the spatial correlation is small and indicated predicted spatial catchment range closest to the hospital.

Model parameters	Busia hospital	Kakamega hospital	Vihiga hospital	Kisumu hospital
Intercept	-7.88 (-8.44 - -7.41)	-8.18 (-8.69 - -7.74)	-7.40 (-0.7.81 - -7.06)	-10.25 (-10.98 - -9.65)
Distance (Euclidean)	-0.01 (-0.11 -0.08)	-0.80 (-0.91 - -0.71)	-0.30 (-0.57 - -0.08)	0.71 (0.46 - 0.91)
Model spatial range (km)	7.76 (6.65 – 9.98)	7.70 (6.61 - 9.90)	3.32 (2.21 – 4.43)	4.40 (3.33 – 6.65)
Marginal variance	3.56 (2.79 - 4.50)	4.66 (3.67 - 6.02)	2.51 (1.80 - 3.48)	22.89 (16.63 - 31.76)
scale parameter	39.6 (30.97 - 50.56)	38.05 (30.47 - 46.27)	105.39 (73.28 - 150.23)	63.72 (48.22 - 84.15)

References

1 Rue, H. & Held, L. *Gaussian Markov Random Fields: Theory and Applications (Chapman & Hall/CRC Monographs on Statistics & Applied Probability).* (Chapman and Hall/CRC, 2005).

2 Cressie, N. Fitting variogram models by weighted least squares. *Mathematical Geology* **17**, 563-586, doi:10.1007/bf01032109 (1985).

3 Cressie, N. Kriging Nonstationary Data. *Journal of the American Statistical Association* **81**, 625-634 (1986).

4 Rue, H., Martino, S. & Chopin, N. Approximate Bayesian inference for latent Gaussian models by using integrated nested Laplace approximations. *Journal of the Royal Statistical Society: Series B (Statistical Methodology)* **71**, 319-392, doi:10.1111/j.1467-9868.2008.00700.x (2009).

5 Kloek, T. & Dijk, H. K. v. Bayesian Estimates of Equation System Parameters: An Application of Integration by Monte Carlo. *Econometrica* **46**, 1-19, doi:10.2307/1913641 (1978).

6 Schrödle, B. & Held, L. A primer on disease mapping and ecological regression using INLA. *Computational Statistics* **26**, 241-258, doi:10.1007/s00180-010-0208-2 (2010).

7 Rue, H. v. & Martino, S. Approximate Bayesian inference for hierarchical Gaussian Markov random field models. *Journal of Statistical Planning and Inference* **137**, 3177-3192 (2007).

8 Fahrmeir, L. & Lang, S. Bayesian inference for generalized additive mixed models based on Markov random field priors *Journal of the Royal Statistical Society, Series C* **2**, 201-220 (2001).

9 Gneiting, T. & Raftery, A. E. Strictly Proper Scoring Rules, Prediction, and Estimation. *Journal of the American Statistical Association* **102**, 359-378, doi:10.1198/016214506000001437 (2007).

10 Czado, C., Gneiting, T. & Held, L. Predictive model assessment for count data. *Biometrics* **65**, 1254-1261, doi:10.1111/j.1541-0420.2009.01191.x (2009).

11 Spiegelhalter, D. J., Best, N. G., Carlin, B. P. & Van Der Linde, A. Bayesian measures of model complexity and fit. *Journal of the Royal Statistical Society: Series B (Statistical Methodology)* **64**, 583-639, doi:10.1111/1467-9868.00353 (2002).
